# Asarinin Inhibits RANKL-Induced Osteoclast Differentiation by Targeting the p38/ERK–c-Fos–NFATc1 Axis

**DOI:** 10.3390/ijms27167159

**Published:** 2026-08-11

**Authors:** Lifang Zhang, Chengxu Xie, Xinyi Bao, Xiaohan Li, Heriberto Velez, Farwa Basit, Yili Ding, Mojtaba Tabandeh, Vishwa Deepak

**Affiliations:** 1 Osteoimmunology & Drug Discovery Research Group, Department of Biology, College of Science, Mathematics and Technology, Wenzhou-Kean University, 88 Daxue Road, Wenzhou 325060, China; 2 Dorothy and George Hennings College of Science, Mathematics and Technology, Kean University, 1000 Morris Ave, Union, NJ 07083, USA; 3 Department of Chemistry, College of Science, Mathematics and Technology, Wenzhou-Kean University, 88 Daxue Road, Wenzhou 325060, China; 4 International Frontier Interdisciplinary Research Institute (IFIRI) of Wenzhou-Kean University, Wenzhou 325060, China; 5 Wenzhou Municipal Key Laboratory for Applied Biomedical and Biopharmaceutical Informatics, Wenzhou-Kean University, Wenzhou 325060, China; 6 Zhejiang Bioinformatics International Science and Technology Cooperation Center, Wenzhou-Kean University, Wenzhou 325060, China; 7 Zhejiang-Malaysia Joint Laboratory for Rare Medicinal Resources, Wenzhou-Kean University, 88 Daxue Road, Wenzhou 325060, China

**Keywords:** asarinin, osteoclast, RANKL, c-Fos, NFATc1

## Abstract

Excessive osteoclast formation is a key contributor to pathological bone loss in disorders such as osteoporosis and rheumatoid arthritis. Asarinin, a natural lignan, has not previously been examined in the context of osteoclast differentiation. Here, we investigated the anti-osteoclastogenic effects of asarinin using RANKL-stimulated RAW264.7 cells. Asarinin significantly suppressed TRAP-positive multinucleated osteoclast formation. Mechanistically, asarinin selectively inhibited RANKL-induced phosphorylation of p38 and ERK MAPKs, leading to reduced c-Fos expression and inhibition of NFATc1 activation. In addition, asarinin disrupted actin ring formation in mature osteoclasts. Collectively, these findings identify asarinin as a pathway-selective inhibitor of osteoclast differentiation, acting through a mechanism consistent with modulation of the p38/ERK–c-Fos–NFATc1 signaling cascade while sparing parallel signaling pathways.

## 1. Introduction

Osteoclasts are multinucleated cells responsible for bone resorption, and their excessive activity leads to pathological bone loss in conditions such as osteoporosis, rheumatoid arthritis, and periodontal disease [1]. The receptor activator of nuclear factor-κB ligand (RANKL) is the primary cytokine that induces osteoclast differentiation by binding to its receptor RANK on osteoclast precursors [2]. RANKL-RANK interaction activates multiple downstream signaling cascades, including mitogen-activated protein kinases (MAPKs) such as p38, ERK, and JNK, as well as the NF-κB pathway [3]. These pathways converge to induce critical transcription factors including c-Fos and NFATc1. c-Fos, a component of the AP-1 complex, cooperates with NFATc1, the master transcription factor for osteoclast differentiation, to regulate osteoclast-specific gene expression [4].

Natural compounds with anti-osteoclastogenic properties have gained attention as potential alternatives to conventional bone resorption therapies due to their favorable safety profiles [5]. Importantly, pathway-selective modulators that target specific signaling cascades while preserving others may offer therapeutic advantages by minimizing compensatory mechanisms and off-target effects. Recent studies have identified novel molecular regulators of osteoclast differentiation, including intraflagellar transport proteins that modulate RANKL signaling [6]. Asarinin is a lignan isolated from *Asarum* species and has demonstrated anti-inflammatory and neuroprotective activities [7]. Notably, asarinin has been reported to modulate intracellular signaling pathways associated with inflammatory and stress responses, suggesting potential activity in MAPK-dependent transcriptional networks relevant to osteoclast differentiation [8]. However, its effects on osteoclast differentiation and pathway selectivity have not been investigated. This study aimed to evaluate the anti-osteoclastogenic activity of asarinin and elucidate its pathway-specific molecular mechanisms using RANKL-induced RAW264.7 cells as an in vitro model.

## 2. Results and Discussion

**Figure 1:**
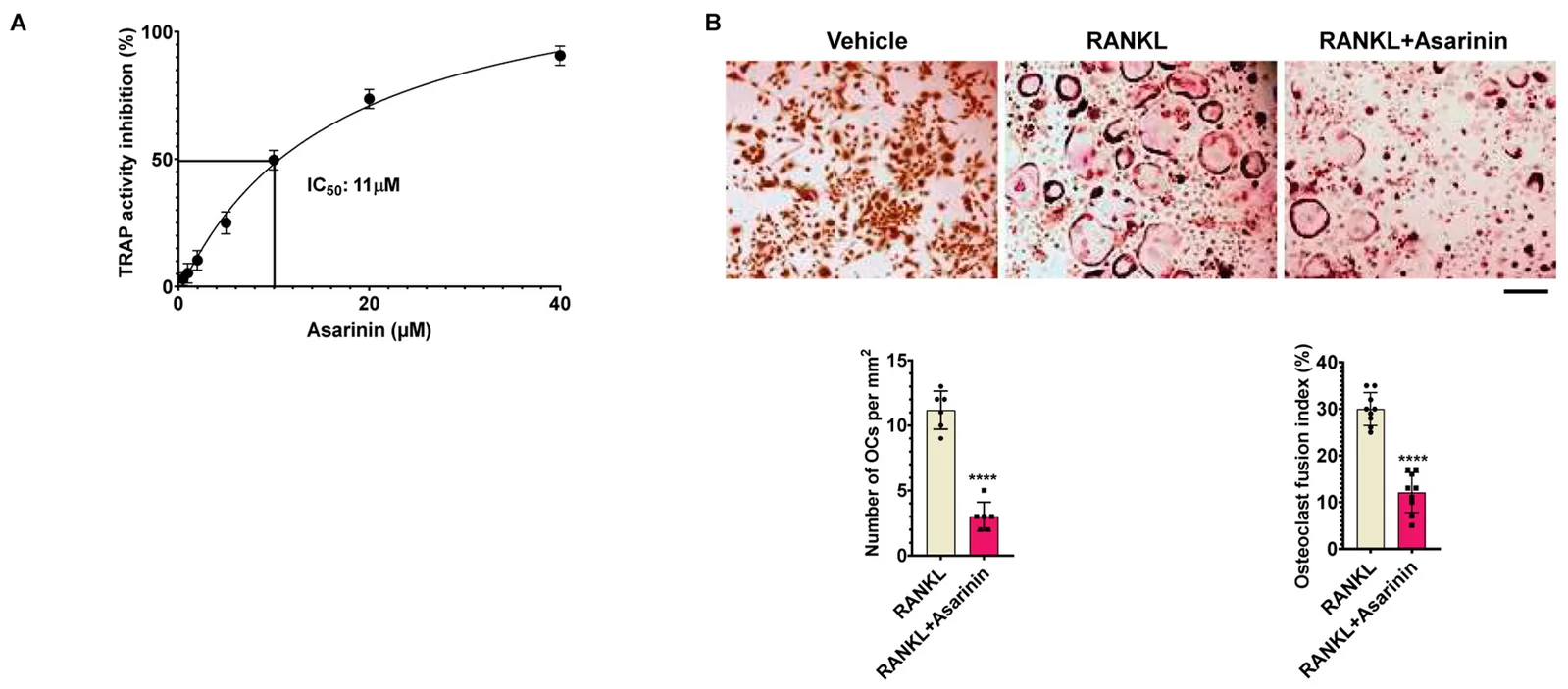
**Asarinin inhibits RANKL-induced osteoclast formation in a dose-dependent manner.** (**A**) RAW264.7 cells were treated with RANKL (50 ng/mL) in the presence of increasing concentrations of asarinin (0.5–40 μM) for 5 days, and TRAP activity was quantified to generate a dose–response inhibition curve. Asarinin inhibited RANKL-induced TRAP activity in a concentration-dependent manner, with a calculated half-maximal inhibitory concentration (IC50) of 11 μM (95% CI, 10.2–12.3 μM); *N* = 3. The concentration of 10 μM, used in all subsequent experiments, was selected on this basis as it approximates the IC50 for osteoclast inhibition. (**B**) RAW264.7 cells were cultured with RANKL (50 ng/mL) and treated with vehicle or asarinin (10 μM) for 5 days. Cells were fixed and stained for TRAP activity. Representative images show TRAP-positive multinucleated osteoclasts (purple). Osteoclasts were defined as TRAP^+^ cells containing ≥3 nuclei. Vehicle-treated cells did not form TRAP-positive multinucleated osteoclasts under these conditions and were confirmed non-toxic by CCK-8 assay (Supplementary Figure S1); statistical comparison was therefore restricted to RANKL versus RANKL + asarinin, consistent with standard practice in the osteoclastogenesis literature. Bar graphs show quantification of osteoclast number per mm^2^ and fusion index (%). Scale bar, 100 μm. Data represent mean ± SD; *N* = 6. **** *p* < 0.0001 vs. RANKL alone.

**Asarinin Suppresses RANKL-Induced Osteoclast Formation.** To evaluate whether asarinin affects osteoclast differentiation, RAW264.7 cells were treated with RANKL in the presence of increasing concentrations of asarinin. Asarinin inhibited RANKL-induced TRAP activity in a concentration-dependent manner, with a calculated IC50 of 11 μM (95% CI, 10.2–12.3 μM) (Figure 1A). Based on this dose–response relationship, 10 μM was selected for subsequent experiments. TRAP staining revealed that RANKL stimulation induced robust formation of TRAP-positive multinucleated osteoclasts in vehicle-treated controls (Figure 1B). In contrast, asarinin (10 μM) treatment markedly reduced both the number of mature osteoclasts and the osteoclast fusion index (Figure 1B). No overt changes in cell morphology or attachment were observed under the tested conditions. Asarinin had no effect on cell viability across the differentiation timeframe tested (Supplementary Figure S1). These results indicate that asarinin suppresses RANKL-induced osteoclast differentiation in a dose-dependent manner.

**Figure 2:**
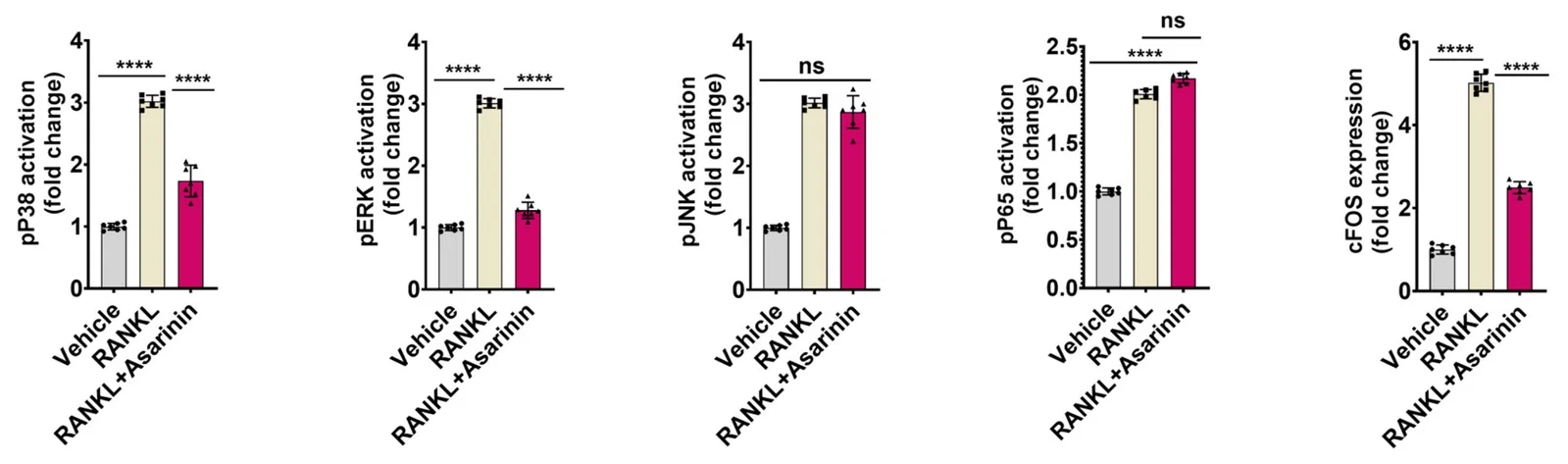
**Asarinin selectively suppresses p38/ERK signaling, leading to reduced c-Fos expression.** RAW264.7 cells were treated with vehicle, RANKL (50 ng/mL), or RANKL plus asarinin (10 µM) for 10 min to assess phosphorylation of p38, ERK, JNK, and p65 (NF-κB), or for 24 h to assess c-Fos expression. All endpoints were quantified by ELISA. Asarinin significantly reduced phosphorylation of p38 and ERK, and markedly suppressed RANKL-induced c-Fos expression, whereas phosphorylation of JNK and p65 was not significantly affected relative to RANKL alone. Bar graphs show fold change relative to vehicle, normalized to total protein. Data represent mean ± SD; *N* = 7. **** *p* < 0.0001; ns, not significant.

**Asarinin Exhibits Pathway Selectivity by Ameliorating p38/ERK While Preserving JNK and NF-κB Signaling.** To understand the molecular mechanism underlying asarinin’s anti-osteoclastogenic activity, we examined its effects on RANKL-activated signaling pathways. Asarinin markedly suppressed RANKL-induced phosphorylation of p38 and ERK MAPKs (Figure 2). In contrast, JNK phosphorylation and NF-κB activation (p65 phosphorylation) remained largely unaffected by asarinin treatment (Figure 2), demonstrating clear pathway-specific modulation. This selective downregulation of p38 and ERK, while preserving JNK and NF-κB pathways, is particularly noteworthy as it suggests that asarinin acts through a specific molecular mechanism rather than general kinase inhibition. Such pathway selectivity may be therapeutically advantageous, as complete blockade of all RANKL-activated pathways could trigger compensatory mechanisms or unwanted side effects.

**Figure 3:**
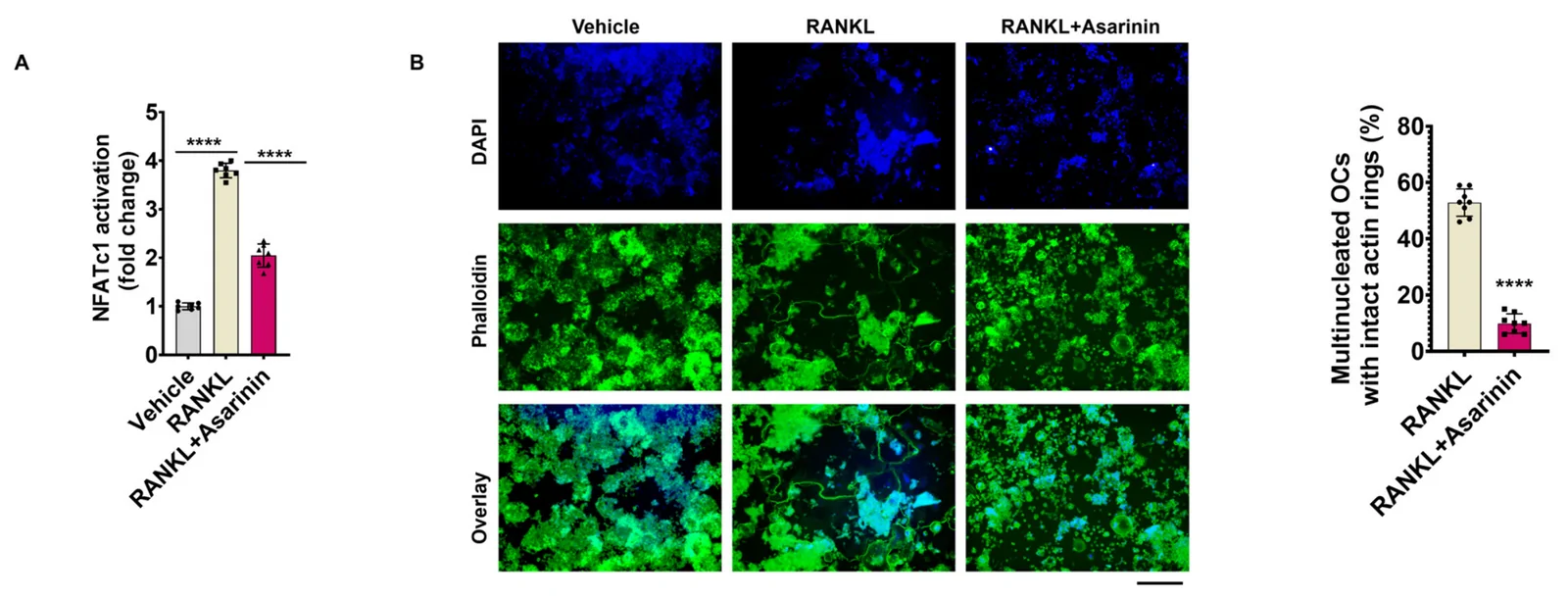
**Asarinin inhibits NFATc1 activation and disrupts actin ring formation.** (**A**) RAW264.7 cells were treated with RANKL (50 ng/mL) in the presence or absence of asarinin (10 μM) for 3 days, and NFATc1 activation was quantified by ELISA in nuclear extracts. The bar graph shows fold change in NFATc1 activation relative to vehicle ± SD; *N* = 7. (**B**) RAW264.7 cells were differentiated with RANKL in the presence or absence of asarinin (10 μM) for 5 days. F-actin (green) was visualized by phalloidin staining and nuclei (blue) were counterstained with DAPI. Representative images show DAPI, phalloidin, and merged overlay channels separately for each condition. Bar graph shows quantification of multinucleated osteoclasts with intact actin rings (%). Scale bar, 50 μm. Data represent mean ± SD; *N* = 8. **** *p* < 0.0001 vs. RANKL alone.

**Asarinin Suppresses c-Fos Expression and Prevents NFATc1 Nuclear Translocation.** c-Fos is a key transcription factor induced predominantly by p38 and ERK MAPK signaling and is essential for NFATc1 induction during osteoclastogenesis [9]. Consistent with the selective inhibition of p38 and ERK, ELISA-based quantification revealed that asarinin significantly reduced c-Fos expression in RANKL-stimulated cells, from a 5.0-fold induction to a 2.5-fold induction relative to vehicle (Figure 2), demonstrating the functional consequence of upstream MAPK modulation. NFATc1 is the master transcription factor that regulates osteoclast-specific genes, and its activation is a prerequisite for osteoclast differentiation [10]. Quantification of nuclear NFATc1 activity revealed that RANKL stimulation induced robust NFATc1 activation in control cells, corresponding to an approximately 4-fold increase relative to vehicle (Figure 3A). In contrast, asarinin treatment significantly inhibited NFATc1 activation, reducing it to approximately 2.0-fold over vehicle (Figure 3A). This suppression of NFATc1 activation, coupled with c-Fos suppression, is consistent with a model in which p38/ERK modulation contributes to asarinin’s anti-osteoclastogenic effect via the p38/ERK–c-Fos–NFATc1 signaling cascade. As these measurements are correlative, direct causal confirmation of this pathway (e.g., via rescue experiments using a constitutively active MEK1 or a p38 activator) is identified as a priority for future work (see Limitations).

**Asarinin Disrupts Actin Ring Formation in Mature Osteoclasts.** Functional osteoclasts require the formation of F-actin–rich sealing zones, or actin rings, to support bone resorption [11]. Phalloidin staining demonstrated prominent actin ring structures in RANKL-induced osteoclasts (Figure 3B). In contrast, asarinin treatment significantly reduced the proportion of multinucleated cells exhibiting intact actin rings (Figure 3B), indicating impaired osteoclast maturation and cytoskeletal organization. Disruption of actin ring formation further supports a functional inhibitory effect of asarinin on osteoclast development.

**Pathway Selectivity in Broader Context.** The selective suppression of p38 and ERK signaling, without measurable effects on JNK or NF-κB activation, distinguishes asarinin from pan-MAPK inhibitors and from several previously characterized anti-osteoclastogenic natural compounds that act more broadly across RANKL-activated pathways [12,13]. This selectivity is consistent with a growing body of evidence suggesting that individual MAPK branches contribute non-redundantly to osteoclast differentiation, with p38 and ERK converging more directly on c-Fos and AP-1-dependent transcription, while JNK and NF-κB additionally support other RANKL-dependent processes such as cell survival [14]. Whether this selectivity reflects a specific interaction between asarinin and an upstream component of the p38/ERK cascade (e.g., MKK3/6 or MEK1/2), or a more indirect effect on cellular redox state or receptor-proximal signaling, remains to be determined. Identifying the direct molecular target of asarinin would clarify whether its pathway selectivity is a generalizable feature of this lignan class or specific to the concentration and cellular context tested here.

Several limitations should be considered when interpreting these findings. First, experiments were conducted exclusively in the RAW264.7 murine macrophage cell line; although a dose–response relationship was established (Figure 1A), confirmation in primary bone marrow-derived macrophages or human osteoclast precursors has not yet been performed. Second, this study is limited to in vitro differentiation assays; functional bone resorption was not directly assessed (e.g., via resorption pit assays on bone or hydroxyapatite-coated substrates), so the extent to which reduced actin ring formation translates into diminished resorptive capacity remains to be confirmed. Third, while p38/ERK modulation, c-Fos suppression, and NFATc1 inhibition were consistently observed together, causal dependence between these downstream events was not directly tested; rescue experiments (e.g., using a constitutively active MEK1 or a p38 activator to restore p38/ERK activity and determine whether this reverses c-Fos/NFATc1 suppression and asarinin’s anti-osteoclastogenic effect) have not yet been performed. Fourth, and separately, the *upstream* molecular target through which asarinin itself modulates p38/ERK signaling has not been identified; whether asarinin acts on MKK3/6, MEK1/2, or a more indirect receptor-proximal or redox-related mechanism remains unknown, and the basis for its pathway selectivity is therefore inferred rather than directly demonstrated.

Future studies should address these gaps by (1) confirming asarinin’s anti-osteoclastogenic activity in primary osteoclast precursor populations, (2) performing functional resorption assays to directly link the observed cytoskeletal changes to resorptive activity, (3) conducting rescue experiments (e.g., constitutively active MEK1 or a p38 activator) to establish direct causal dependence of asarinin’s anti-osteoclastogenic effect on p38/ERK modulation, (4) using kinase activity assays or binding studies to identify the direct molecular target(s) of asarinin upstream of p38/ERK, and (5) evaluating asarinin’s efficacy and safety in relevant in vivo models of pathological bone loss, such as ovariectomized or inflammatory bone-loss mouse models. Together, these efforts would help establish whether the pathway-selective activity observed here translates into a therapeutically meaningful and safe approach for managing osteoclast-mediated bone diseases.

## 3. Materials and Methods

**Cell Culture and Reagents.** Murine macrophage RAW264.7 cells were cultured in Dulbecco’s Modified Eagle Medium (DMEM; Gibco, Grand Island, NY, USA) supplemented with 10% fetal bovine serum and 1% penicillin–streptomycin at 37 °C in a humidified incubator with 5% CO_2_. Asarinin (purity ≥99%; CAS No. 133-04-0; HY-N0701) was purchased from MedChemExpress (Monmouth Junction, NJ, USA). Recombinant mouse RANKL (462-TEC-010) was obtained from R&D Systems (Minneapolis, MN, USA).

**Cell Viability Assay.** RAW264.7 cells were seeded in 96-well plates (5 × 10^3^ cells/well) and treated with vehicle or asarinin (10 μM) for up to 4 days. Cell viability was assessed using a Cell Counting Kit-8 (CCK-8; Service Bio, Wuhan, China) according to the manufacturer’s protocol. Cell viability was expressed relative to vehicle-treated controls at each timepoint.

***TRAP Activity Assay.*** RAW264.7 cells were seeded in 96-well plates at 3 × 10^3^ cells per well. After overnight attachment, the cells were treated with RANKL at 50 ng/mL in the presence of asarinin (0.5–40 μM; seven-point concentration series). Treatment continued for 3 days, and the culture medium was replaced every 48 h. Tartrate-resistant acid phosphatase (TRAP) activity was measured using a p-nitrophenyl phosphate-based colorimetric assay. Cells were lysed with 0.1% Triton X-100. The lysates were incubated at 37 °C with acetate buffer containing p-nitrophenyl phosphate and sodium tartrate. The release of p-nitrophenol was measured at 405 nm using a microplate reader. TRAP activity was normalized to the vehicle control, and percent inhibition at each asarinin concentration was calculated relative to the RANKL-alone control. The half-maximal inhibitory concentration (IC_50_) was determined by nonlinear regression (log[inhibitor] vs. normalized response) using GraphPad Prism version 8.0 (GraphPad Software, San Diego, CA, USA).

**TRAP Staining and Osteoclast Quantification.** RAW264.7 cells were seeded in 96-well plates (5 × 10^3^ cells/well) and stimulated with RANKL (50 ng/mL) in the presence or absence of asarinin (10 μM) for 5 days. The culture medium containing RANKL and asarinin was refreshed every 2 days. Cell morphology and attachment were monitored microscopically throughout the differentiation period.

After differentiation, cells were fixed with 4% paraformaldehyde for 15 min and stained for tartrate-resistant acid phosphatase (TRAP) activity using a commercial TRAP staining kit (Servicebio, Wuhan, China) according to the manufacturer’s protocol. TRAP-positive multinucleated cells (≥3 nuclei) were counted as mature osteoclasts under light microscopy (200× magnification). For each condition, at least five fields per well were analyzed and averaged, and osteoclast density was calculated by normalizing counts to the imaged area and expressed as osteoclasts per mm^2^. Osteoclast fusion index was calculated as the percentage of nuclei in multinucleated cells relative to total nuclei.

**ELISA Analysis of MAPK, NF-κB, and c-Fos Activation.** Cells were lysed in protein lysis buffer containing protease and phosphatase inhibitors, and total protein concentration was determined using a BCA protein assay. Phosphorylation of p38 (Thr180/Tyr182), ERK1/2 (Thr202/Tyr204), JNK (Thr183/Tyr185), and p65 (Ser536) (Jiangsu Enzyme Immunity Industrial Co., Ltd., Yancheng, China), as well as total c-Fos protein levels (Abcam, Waltham, MA, USA), were quantified using commercially available sandwich ELISA kits according to the manufacturers’ protocols. Equal amounts of protein lysate (normalized by BCA assay) were loaded per well and absorbance was measured using a microplate reader. Phospho-protein levels were normalized to total protein levels for the corresponding target where applicable, and all values are expressed as fold change relative to vehicle-treated controls. A validated sandwich ELISA format was selected for these multi-endpoint measurements because it provides fully quantitative, plate-based readouts using kits validated by the manufacturer, enabling higher-throughput screening across multiple signaling targets and treatment conditions in parallel.

**NFATc1 Activation ELISA.** NFATc1 activation was quantified using a commercially available ELISA kit (ELK Biotech, Sugar Land, TX, USA) according to the manufacturer’s protocol. RAW264.7 cells were treated with RANKL (50 ng/mL) in the presence or absence of asarinin (10 µM) for 3 days prior to nuclear extraction. Nuclear extracts were prepared from RAW264.7 cells treated with RANKL in the presence or absence of asarinin, and NFATc1 activity was measured by absorbance and expressed as fold change relative to vehicle-treated controls.

**Immunofluorescence Microscopy.** For actin ring visualization, cells cultured on glass coverslips were fixed with 4% paraformaldehyde, permeabilized with 0.1% Triton X-100, blocked with 3% BSA, and stained with Alexa Fluor 488-conjugated phalloidin. Nuclei were counterstained with DAPI. Images were captured using a Keyence microscope. All images were acquired using identical exposure settings within each experiment. The percentage of multinucleated osteoclasts with intact actin rings was quantified from at least five random fields.

**Statistical Analysis.** For comparisons involving two groups, statistical significance was determined using Student’s *t*-test. For comparisons involving three groups, one-way analysis of variance (ANOVA) followed by Tukey’s post hoc test was used. Each experiment was performed in three independent biological replicates, with two to three technical replicates (wells or fields) per condition within each independent experiment; the *N* reported in each figure legend reflects the total number of data points pooled across all three independent experiments. *p* < 0.05 was considered statistically significant.

## 4. Conclusions

In conclusion, this study demonstrates that asarinin is a pathway-selective inhibitor of RANKL-induced osteoclastogenesis, acting through multiple coordinated mechanisms: (1) selective suppression of p38 and ERK MAPK signaling while preserving JNK and NF-κB pathways, (2) reduction of c-Fos expression, (3) inhibition of NFATc1 activation, and (4) disruption of actin ring formation. This pathway-selective profile distinguishes asarinin from non-selective kinase inhibitors and suggests a targeted mode of action that may confer therapeutic advantages by minimizing compensatory signaling and off-target effects.

## Data Availability

The authors confirm that all data generated during this study are included in this published article.

## Supplementary Materials

The following supporting information can be downloaded at: https://www.mdpi.com/article/10.3390/ijms27167159/s1.
