# Supplementary material for: Asarinin Inhibits RANKL-Induced Osteoclast Differentiation by Targeting the p38/ERK–c-Fos–NFATc1 Axis
Source: Int J Mol Sci. 2026 Aug 11;27(16):7159. doi: 10.3390/ijms27167159 (PMC13512931; doi:10.3390/ijms27167159)
Supplement: Supplementary file 1 [file ijms-27-07159-s001.zip › ijms-4469795-supplementary.pdf]

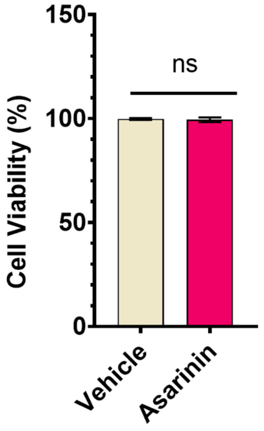

Supplementary Figure S1. Asarinin does not affect cell viability. RAW264.7 cells were treated with vehicle or asarinin (10 μM) for 48 h, and cell viability was assessed by CCK-8 assay. Data are expressed as mean ± SD from three independent experiments and normalized to vehicle-treated controls (100%). ns, not significant (Student's t-test).
